# Expression of the Peroxisome Proliferator-Activated Receptors (PPARs) in the Hepatic Stellate Cells

**DOI:** 10.1186/1476-5926-2-S1-S17

**Published:** 2004-01-14

**Authors:** Takeya Sato, Mitsuru Sato, Mitsutaka Miura, Nobuyo Higashi, Da-Ren Wang, Shinsuke Suzuki, Katsuyuki Imai, Naosuke Kojima, Haruki Senoo

**Affiliations:** 1 Department of Anatomy, Akita University School of Medicine, Akita, 010-8543, Japan

## Introduction

Hepatic stellate cells (HSCs) have an important role in maintaining vitamin A (retinoid) homeostasis [1,2]. Peroxisome proliferator-activated receptors (PPARs) are members of the steroid/retinoid nuclear hormone receptor superfamily of ligand-activated transcription factors, form a heterodimer with retinoid X receptor (RXR), and play an important role in lipid metabolisms. Several reports [3-5] have suggested that expression of PPAR-gamma was reduced with the acquisition of activated phenotype (termed as "activation") such as lack of cytoplasmic lipid droplets containing vitamin A in subcultured HSC. Previously, we showed that the subcultured HSCs restored the cytoplasmic lipid droplets emanating vitamin A autofluorescence by the addition of retinyl acetate to culture medium [6]. However, it remains unclear whether or not PPAR-gamma expression is involved in formation of vitamin A-containing lipid droplets and maintaining of vitamin A homeostasis in HSCs. In this study, we examined roles of PPARs, particularly PPAR-gamma, in accumulation of vitamin A-containing lipid droplets in cultivated HSCs.

## Methods

Isolation and cultivation of rat HSCs was performed as previously described [6]. An adipocyte cell line, 3T3-L1, was purchased from Japanese Collection of Research Bioresources (JCRB, Osaka, Japan) and cell culture was carried out according to the supplier's protocol. HSCs were cultured with or without 10 micromolar retinyl acetate and 10 micromolar PPAR-gamma ligand such as ciglitazone. Autofluorescence of vitamin A emanated from the cytoplasmic lipid droplets in HSCs was monitored as previously described [6]. Total RNAs were extracted from the cultured HSCs and 3T3-L1 adipocytes using TRIZOL reagent and reverse-transcribed with MMLV reverse transcriptase. The resultant cDNAs were used for RT-PCR analysis with a specific primer pair for each PPAR subtype, as well as a primer pair for glyceraldehyde-3-phosphate dehydrogenase (G3PDH) as an internal control.

## Results

**Figure 1:**
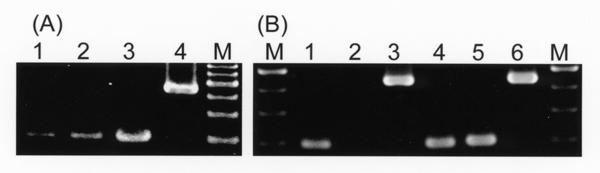
RT-PCR analysis for expression of PPAR subtypes (A) and PPAR-gamma isoform (B) in HSCs. Total RNA was isolated from HSCs (lanes 1-4 in Fig. 1-A and lanes 1-3 in Fig. 1-B) or 3T3-L1 adipocytes (lanes 4-6 in Fig. 1-B). (A): Total RNA was reverse transcribed, and then the cDNAs were amplified by PCR using a specific primer pair for PPAR-alpha (lane 1), PPAR-delta (lane 2), and PPAR-gamma (lane 3), as well as a primer pair for G3PDH as an internal control (lane 4). (B): Expression of mRNAs for PPAR-gamma isoform in HSCs was analyzed with the common primer pair for both PPAR gamma 1 and gamma 2 isoforms (lanes 1 and 4) and the specific primer pair for PPAR-gamma 2 isoform (lanes 2 and 5). Amplification of G3PDH cDNA was used as an internal control (lanes 3 and 6). A single band with the predicted size, 200 bp for each of PPAR-gamma isoforms and 460 bp for G3PDH was detected. M: DNA size markers.

RT-PCR analysis showed that mammalian PPAR-alpha, delta and gamma (Fig. 1-A) but not adipocyte specific PPAR-gamma 2 (Fig. 1-B) were expressed in cultured HSCs, and the subtype PPAR-gamma predominantly expressed (Fig. 1-A). Among PPAR-gamma isoforms, HSCs expressed PPAR-gamma 1.

**Figure 2:**
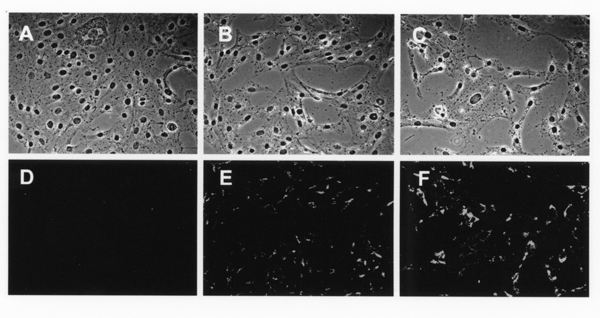
Increase of vitamin A-containing lipid droplets by addition of PPAR-gamma agonist in cultured HSCs. HSCs were cultured for 48 h in the presence of (A and D) 0.2% ethanol used as vehicle, or (B and E) 10 micromolar retinyl acetate, or (C and F) both 10 micromolar retinyl acetate and 10 micromolar PPAR-gamma agonist, ciglitazone. After fixation of the cells (phase contrast images: A, B, and C), autofluorescence emanated from vitamin A in the cytoplasmic lipid droplets was detected and recorded by using a fluorescence microscope equipped with a chilled CCD camera (D, E, and F).

We next examined the effect of PPAR-gamma on incorporation of vitamin A and formation of vitamin A-containing lipid droplets when the vitamin A was added to the culture medium. When HSCs were cultured without the addition of retinyl acetate, the lipid droplets were not formed in the cytoplasm (Fig. 2-A and 2-D). The cells restored the cytoplasmic lipid droplets emanating vitamin A-autofluorescence after the addition of 10 micromolar retinyl acetate to the culture medium (Fig. 2-B and 2-E). Accumulation of lipid droplets was further enhanced by the simultaneous addition of 10 micromolar retinyl acetate and 10 micromolar PPAR-gamma ligand to the culture medium (Fig. 2-C and 2-F).

## Discussion

In this study, we demonstrated that PPAR-gamma 1 was expressed in HSCs and had a promotional role in vitamin A uptake and lipid droplet formation by addition of its ligand to the culture medium. Transdifferentiation of HSCs occurs following liver injury or merely during cell culture after isolation from the liver tissue, and is accompanied with the loss of cytoplasmic lipid droplets containing vitamin A. Several transcription factors such as NF-kappa B [7], AP-1 [8], and Kruppel-like transcription factors [9] have been described to be upregulated accompanied by acquiring the activated phenotype of HSCs. Conversely, other transcription factors such as Id1 [10] and Ets-1 [11] are active in quiescent HSCs, and these activities are diminished during HSC activation. The latter types of transcription factors may suppress HSC activation and/or be required for maintaining the quiescent phenotype of HSCs. Several reports indicate that the loss of PPAR-gamma expression and transcriptional activity is coupled with HSC activation [3-5]. Taken together, we conclude that PPAR-gamma 1 have a promotional role in vitamin A uptake, lipid droplet formation, and probably maintaining the quiescent vitamin A-storing phenotype of HSCs.

## References

[B1] Blomhoff R, Wake K (1991). Perisinusoidal stellate cells of the liver: important roles in retinol metabolism and fibrosis. FASEB J.

[B2] Blomhoff R, Green MH, Berg T, Norum KR (1990). Transport and storage of vitamin A. Science.

[B3] Galli A, Crabb D, Price D, Ceni E, Salzano R, Surrenti C, Casini A (2000). Peroxisome proliferator-activated receptor – transcriptional regulation is involved in platelet-derived growth factor-induced proliferation of human hepatic stellate cells. Hepatology.

[B4] Miyahara T, Schrum L, Rippe R, Xiong S, Yee HF, Motomura K, Anania FA, Motomura K, Anania FA, Willson TM, Tsukamoto H (2000). Peroxisome proliferator-activated receptors and hepatic stellate cell activation. J Biol Chem.

[B5] Marra F, Efsen E, Romanelli RG, Caligiuri A, Pastacaldi S, Batignani G, Bonacchi A, Caporale R, Laffi G, Pinzani M, Gentilini P (2000). Ligands of peroxisome proliferator-activated receptor gamma modulate profibrogenic and proinflammatory actions in hepatic stellate cells. Gastroenterology.

[B6] Sato M, Kojima N, Miura M, Imai K, Senoo H (1998). Induction of cellular processes containing collagenase and retinoid by integrin-binding to interstitial collagen in hepatic stellate cell culture. Cell Biol Int.

[B7] Lee KS, Buck M, Houglum K, Chojkier M (1995). Activation of hepatic stellate cells by TGF alpha and collagen type I is mediated by oxidative stress and through c-myb expression. J Clin Invest.

[B8] Bahr MJ, Vincent KJ, Arthur MJ, Fowler AV, Smart DE, Wright MC, Clark IM, Benyon RC, Iredale JP, Mann DA (1999). Control of the tissue inhibitor of metalloproteinases-1 promoter in culture-activated rat hepatic stellate cells: regulation by activator protein-1 DNA binding proteins. Hepatology.

[B9] Ratziu V, Lalazar A, Wong L, Dang Q, Collins C, Shaulian E, Jensen S, Friedman SL (1998). Zf9, a Kruppel-like transcription factor up-regulated in vivo during early hepatic fibrosis. Proc Natl Acad Sci.

[B10] Vincent KJ, Jones E, Arthur MJ, Smart DE, Trim J, Wright MC, Mann DA (2001). Regulation of E-box DNA binding during in vivo and in vitro activation of rat and human hepatic stellate cells. Gut.

[B11] Knittel T, Kobold D, Dudas J, Saile B, Ramadori G (1999). Role of the Ets-1 transcription factor during activation of rat hepatic stellate cells in culture. Am J Pathol.

